# Immunohistological Evaluation of Revascularized Immature Permanent Necrotic Teeth Treated by Platelet-Rich Plasma: An Animal Investigation

**DOI:** 10.22074/cellj.2016.4567

**Published:** 2016-08-24

**Authors:** Saeed Moradi, Ali Talati, Maryam Forghani, Amir Hossein Jafarian, Mandana Naseri, Shiva Shojaeian

**Affiliations:** 1 Dental Material Research Center, Department of Endodontics, Faculty of Dentistry, Mashhad University of Medical Sciences, Mashhad, Iran; 2 Cancer Molecular Pathology Research Center, Faculty of Medicine, Mashhad University of Medical Sciences, Mashhad, Iran; 3 Department of Endodontics, Dental School, Shahid Beheshti University of Medical Sciences, Tehran, Iran

**Keywords:** Platelet-Rich Plasma, Revascularization, Vascular Endothelial Growth Factor

## Abstract

**Objective:**

Pulp regeneration within the root canal of necrotic teeth is considered an ideal treatment to allow for continued root development and recover teeth vitality. This study aims to evaluate the inductive effect of platelet-rich plasma (PRP) on expression of angiogenesis factors and pulpal revascularization of immature necrotic teeth.

**Materials and Methods:**

In this experimental animal study, we randomly divided 28 immature premolars from two mixed breed dogs into four groups, two experimental, negative and a positive control. Premolars in negative control group were left intact to develop normally. In the positive control and experimental groups, we removed the pulps and induced pulp necrosis, after which the chambers were sealed. Then, we applied the revascularization protocol in the experimental teeth located in the right quadrant. Two months later, the same protocol was applied to the left quadrant. The root canals were disinfected by irrigation with sodium hypochlorite (NaOCl) solution and application a triple antibiotic past. Following the induction of a blood clot (BC) inside the canal space, the coronal portion of the canals was assigned to either of two experimental groups: group 1 [BC+PRP+ mineral trioxide aggregate (MTA)], group 2 (BC+MTA). Access cavities were sealed with a Glass Ionomer. The jaws that held the teeth were processed for histologic analysis of newly formed tissue and immunohistochemical evaluation according to vascular endothelial growth factor (VEGF) and factor VIII expressions in the canals.

**Results:**

Histological analysis demonstrated no significant difference in the formation of new vital tissue inside the root canals between groups1 (42.8%) and 2 (43.5%, P>0.05). Based on immunohistochemical evaluation, micro-vessel density (MVD) of the granulation tissues in both groups were similar and were higher compared with the normal pulp. We observed strongly positive expressions of VEGF and factor VIII in the stromal and endothelial cells, with severe intensity after one month. Both factors showed downregulation at three months postoperative.

**Conclusion:**

PRP could not increase the formation of new vital tissue. The immunohistochemical results showed that VEGF and factor VIII played a pivotal role in the formation of new vessels inside the root canals of immature, non-vital teeth.

## Introduction

Root canal therapy, is a common dental treatment, that claims to have a high success rate. A major concern in this procedure, however, is evident in treatment of immature teeth with open apices (1). If the pulp becomes necrotic before the root development is completed, the dentin formation and the root growth will arrest. Revascularization is performed to restore vital tissue, continue root development, and increase the length and thickness of the canal walls in an immature permanent non-vital tooth (2). This procedure has recently become an alternative preferable treatment method to traditional apexification (3). Along with a number of studies on tissue engineering, revascularization has been introduced on the basis of revascularization in replanted immature teeth (4). However, there is no standard treatment protocol to encourage the complete regeneration of pulpdentin complex and the restoration of both immunologic function and nervous sense. Three critical components that contribute to the successful accomplishment of regeneration are stem cells that can produce the hard tissue; signaling molecules to regulate cellular stimulation, proliferation, and differentiation; and a three-dimensional physical scaffold to support cell growth and differentiation (5). In the clinical process, acellular approach is frequently applied. The stem cells that remain in the apical root canal or are present around the root (such as apical papilla, periodontal ligament, bone marrow, and blood vessel) are expected to migrate into the canal after evoking periapical bleeding. The migrating stem cells complete the regeneration process if an appropriate sterile environment and scaffold are provided in the root canal, and the growing signals are adequately developed (6). 

Platelet-rich plasma (PRP), a volume of autologous plasma with a higher platelet concentration than baseline, is recommended as a potentially ideal scaffold because it is rich in growth factors (GFs) and form a three-dimensional fibrin matrix that entraps GFs (7,8). Beside the above mentioned factors, it is critical to have blood vessel formation only the cells located 100-200 µm distances from the capillaries can receive the required nutrients through diffusion. Therefore, it is essential to manage rapid revascularization in the canal to enable survival of the stem cells and regenerated tissue (9). Factor VIII and vascular endothelial growth factor (VEGF) are two primary important factors in angiogenesis. VEGF is a glycoprotein that induces endothelial cell proliferation, migration, and survival (10). This glycoprotein also encourages dental pulp stem cells to differentiate into endothelial cells and organize a capillary-like structure *in vitro* (10,11). Although some studies have evaluated the expression of VEGF in human dental pulps, there is no study that has assessed the amount of this factor present in the root canal during revascularization. 

The aim of the present study was to investigate the immunohistological expression of VEGF and factor VIII in new vital tissue within the canals after revascularization using PRP in canine non-vital immature teeth. 

## Materials and Methods

This experimental animal study used 28 immature one/two-rooted premolar teeth from approximately six-month old male mixed breed dogs (n=2). The Animal Ethic Screening Committee of Mashhad University of Medical Sciences, Mashhad, Iran approved the study protocol. Each animals received antifungal and antiparasite treatment, in addition to vaccination during a one month quarantine period. We randomly divided 24 teeth into two experimental groups. Two mandibular first premolars were assigned to either the positive or negative control groups. General anesthesia was induced by intravenous injection of ketamine hydrochloride (10 mg/kg, Rotexmedica, Trittau, Germany) and xylazine hydrochloride (1 mg/kg, Alfasan, Woerden, Holland) 20 minutes after induction of intramuscular sedation by diazepam (0.1 mg/kg Chemia Daro, Tehran, Iran). Anesthesia was maintained with additional doses as needed. Local anesthesia consisted of an injection of 2% lidocaine with epinephrine 1:80000 (DarouPakhsh, Tehran, Iran). 

### Preparation of platelet-rich plasma

We obtained 20 ml blood from internal jugular vein of each dog. Blood was collected in sterile glass tubes that contained citrate solution as the anticoagulant. To prepare PRP, we centrifuged the whole blood at 3500 rpm for 10 minutes. The erythrocytes precipitated out, leaving the plateletrich upper plasma layer. The plasma layer was aspirated and transferred to three vials. All vials were frozen and maintained -20˚C until the treatment session in which they were thawed at room temperature. PRP coagulation was initiated by the addition 0.07 mL of calcium chloride (1 mol/L) and 0.03 mL of thromboplastin enzyme solution to 1 ml of PRP. The tube was shaken at 37˚C to accelerate the coagulation (12). 

### Treatment protocol

We initiated the procedure on the right quadrants then after two months on the left quadrant (13). During the first session, parallel radiographs were taken from the involved teeth by radiograph paralleling devices (Denstply Rinn Elgin, Illinois, USA). In the experimental and positive control groups, an access was prepared from the buccal surface using a round carbide bur (Mani Inc., Japan) in a high speed handpiece. K-files (DentsplyMaillefer, Switzerland) were inserted in the root canals to determine the canal working length by bisecting radiography. The pulps were extirpated using a 35# barbed broach (Dentsply-Maillefer, Switzerland). The canals were slightly and minimally instrumented to the working length to completely remove the pulp tissue. Supragingival plaque was scaled from the dog's teeth and mixed with sterile saline. Sterile sponges were soaked in plaque suspension and placed in the pulp chamber to induce pulp necrosis. Then, the access cavities were sealed with cavisol (Golchai Co., Iran). The positive control group underwent no additional procedure. 

In the second session, after two weeks, we established aseptic condition under general anesthesia. The oral cavity was rinsed with 0.2% chlorhexidine mouthwash (Shahr Daru, Iran). In the experimental groups, after isolation of the necrotic teeth by a rubber dam, the surfaces were disinfected with 0.2% chlorhexidine. Next we removed the temporary restorations and sponges. The root canals were irrigated with 10 mL normal saline and 10 mL of 5.25% sodium hypochlorite (NaOCl, Merck, Germany) with no mechanical instrumentation. There was no evidence of any vital tissue that remained in the root canals. The canals were dried by sterile paper points. A triple antibiotic paste was placed in the canal to within 2 mm shorter than the working length using a lentulo spiral. The paste was a combination of equal parts of ciprofloxacin (500 mg, Amindaro Co., Iran), metronidazole (250 mg, Pars Darou Co., Iran) and minocycline (100 mg, Razak Co., Iran) which were mixed with sterile saline (0.9% sodium chloride) (14). Finally, the access cavities were sealed by cavisol. 

After three weeks, in the third session, the temporary restorations were aseptically removed under general anesthesia. The canals were rinsed in 10 mL of 5.25% NaOCl and 10 mL of saline in order to remove the antibiotic paste. 30# K file was inserted into the canal, 2 mm beyond the apical foramen to induce bleeding. A large paper cone was used to ensure bleeding in the canal and to control the blood clot (BC) formation below the cemento-enamel junction (CEJ). In group one (BC+PRP+MTA group) that consist of 12 teeth and 21 roots, the activated PRP was immediately placed over the BC using a sampler, followed by mineral trioxide aggregate (MTA) over them. In group two (BC+MTA) that consist of 12 teeth and 23 roots, we directly placed ProRoot MTA (Dentsply Tulsa Dental, USA) over the BC. All teeth were sealed with a glass ionom er one hour after placement of wet cotton pellet on the MTA for initial setting. There was no intervention in the negative control group. Postoperative radiographs were taken in the same manner as the initial radiographs. 

### Histological processing

One month after treatment in the left quadrant, the animals were sacrificed by an overdose of sodium thiopental (50 mg/kg, Biochemie, Austria). They were perfused by 10.0% buffered formalin through cannulated carotid arteries. 

The jaws were resected and each involved tooth was separated as a block section. After removal of soft tissue and excess hard tissue, the blocks were coded and placed in 10.0% formalin for 10 days. The specimens were subsequently decalcified by 14% EDTA. The solution was renewed twice a week for four months. Then, samples were embedded in paraffin and longitudinally cut into 4 µm thick sections. The sections were stained by hematoxylin and eosin (H&E), and evaluated in a blind manner. Samples were evaluated under a light microscope for the presence or absence of fibro-connective tissue and blood vessels in the root canal. 

### Immunohistochemistry

We performed Immunohistochemistry analyses to evaluate regeneration. After deparaffinization and hydration of the slides, they were placed in a microwave oven for antigenic retrieval by incubation in a 1.0% molar citrate buffer (pH=6) for 12 minutes. The immunoperoxidase streptavidin biotin procedure conducted by incubation at the room temperature by 3.0% hydrogen peroxide for 10 minutes and an antibody against VEGF and factor VIII for 60 minutes (Dako Cytomation Inc., Glostrup, Denmark). Finally, streptavidin peroxidase (DAKOLSAB 25 system, peroxidase kit, Dako, Denmark) and chromogen (diaminobenzidine hydrochloride) were applied, respectively. 

Counterstaining was done by Mayer’s hematoxylin. VEGF and factor VIII expression were evaluated as the quantitative (q) and intensity (I) scores. The quantitative score was defined as the percentage of positive cells, whereas the intensity score (I) was classified in four categories based on staining intensity: 1 (negative), 2 (weak), 3 (moderate), and 4 (severe). Microvessel density (MVD) was evaluated based on VEGF and factor VIII immunostaining of thin-walled blood vessels or isolated endothelial cells. We counted our microscopic fields (×400) were and the average number of microvessels was calculated in all fields (15). 

**Fig.1:**
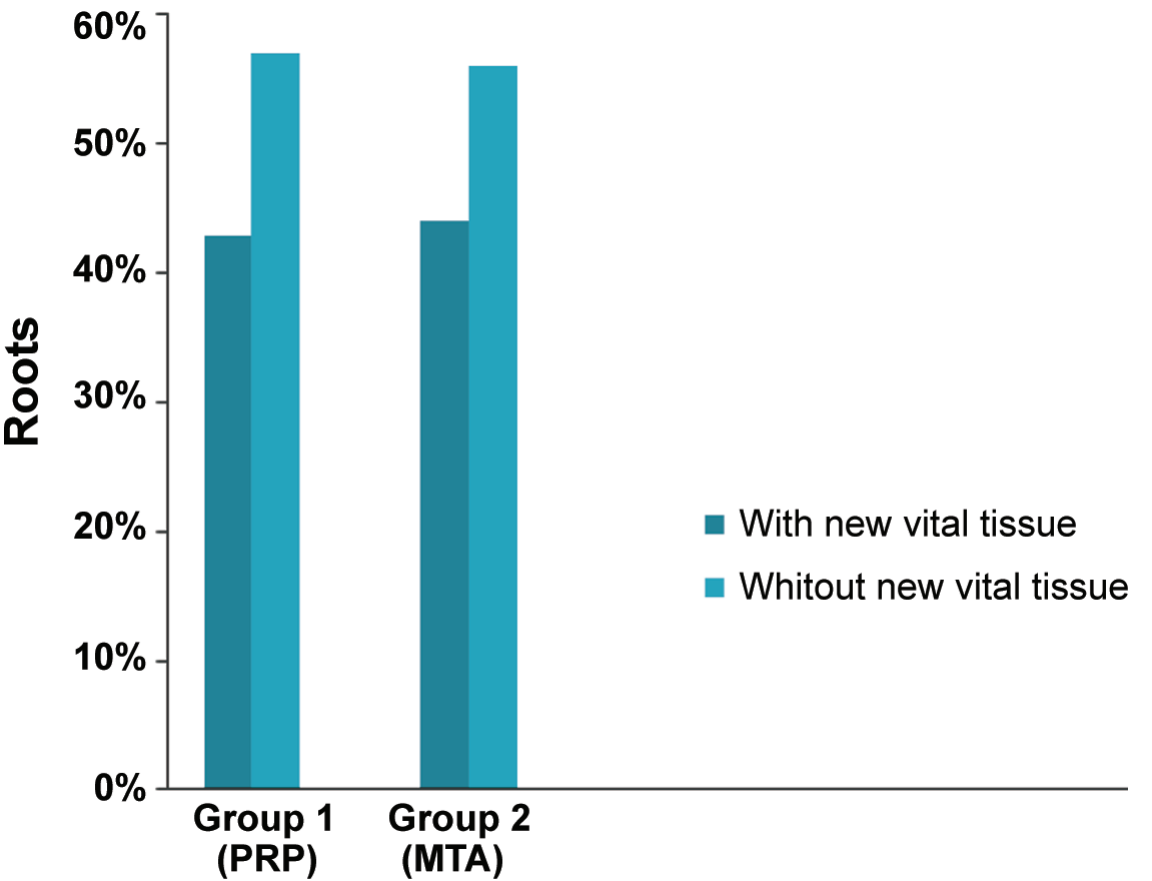
Percent of roots with and without new vital tissue within the canal space in experimental groups evaluated histologically. MTA; Mineral trioxide aggregate and PRP; Platelet-rich plasma.

### Results

**Fig.2:**
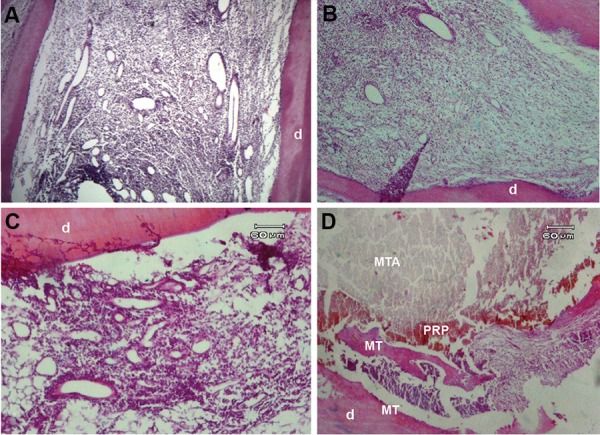
Hematoxylin and eosin (H&E) staining of an immature dog tooth after the revascularization procedure. A. The newly formed tissue contains connective tissue and blood vessels that filled the root canal space with high inflammatory infiltration after one month (original magnification: ×40), B., C. The newly formed tissue with moderate infiltration after 3 months (original magnification: ×40 and ×100), and D. The apposition of mineralized tissue (MT) adjacent to the dentin wall (original magnification: ×100). d; Dentin, MTA; Mineral trioxide aggregate, and PRP; Platelet-rich plasma.

There were newly formed vital tissues observed within the root canals in 42.8% of samples in the BC+PRP+MTA group and in 43.5% of the samples in the BC+MTA group (Fig.1). The chi-square test revealed no significant difference between the two groups (P>0.05). Based on the histological observations, the newly formed tissues included soft connective tissues, vessels and hard mineralized tissues (Fig.2). There were no new vital tissue observed inside the root canals in the positive control group. 

**Fig.3:**
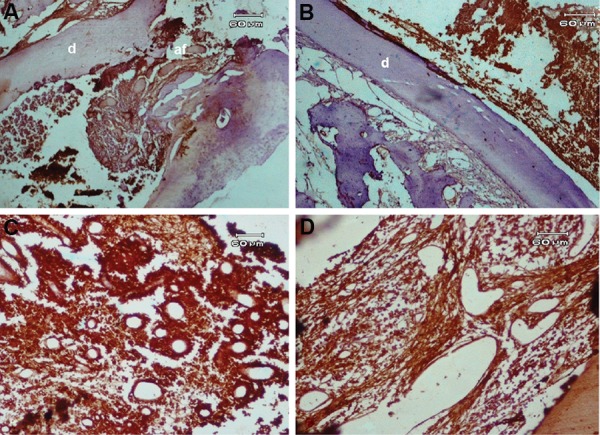
Immunohistochemical staining with angiogenic factors. A. Immunostaining to evaluate the degree of angiogenesis in the root canal (original magnification: ×40), B. Expression of vascular endothelial growth factor (VEGF) in new vital tissue observed in the canal space (original magnification: ×40), C. High expression of VEGF in stromal and endothelial cells of the blood vessel walls. The intensity score was severe (I=4) (original magnification: ×100), and D. Expression of factor VIII in regenerated tissue observed in the canal space (I=3) (original magnification: ×100). d; Dentin.

Based on the results of IHC evaluation, MVD of newly formed vital tissues in groups 1 and 2 were similar (MVD=14) and higher compared to the normal pulp (MVD=6, Fig.3A, B). 

**Fig.4:**
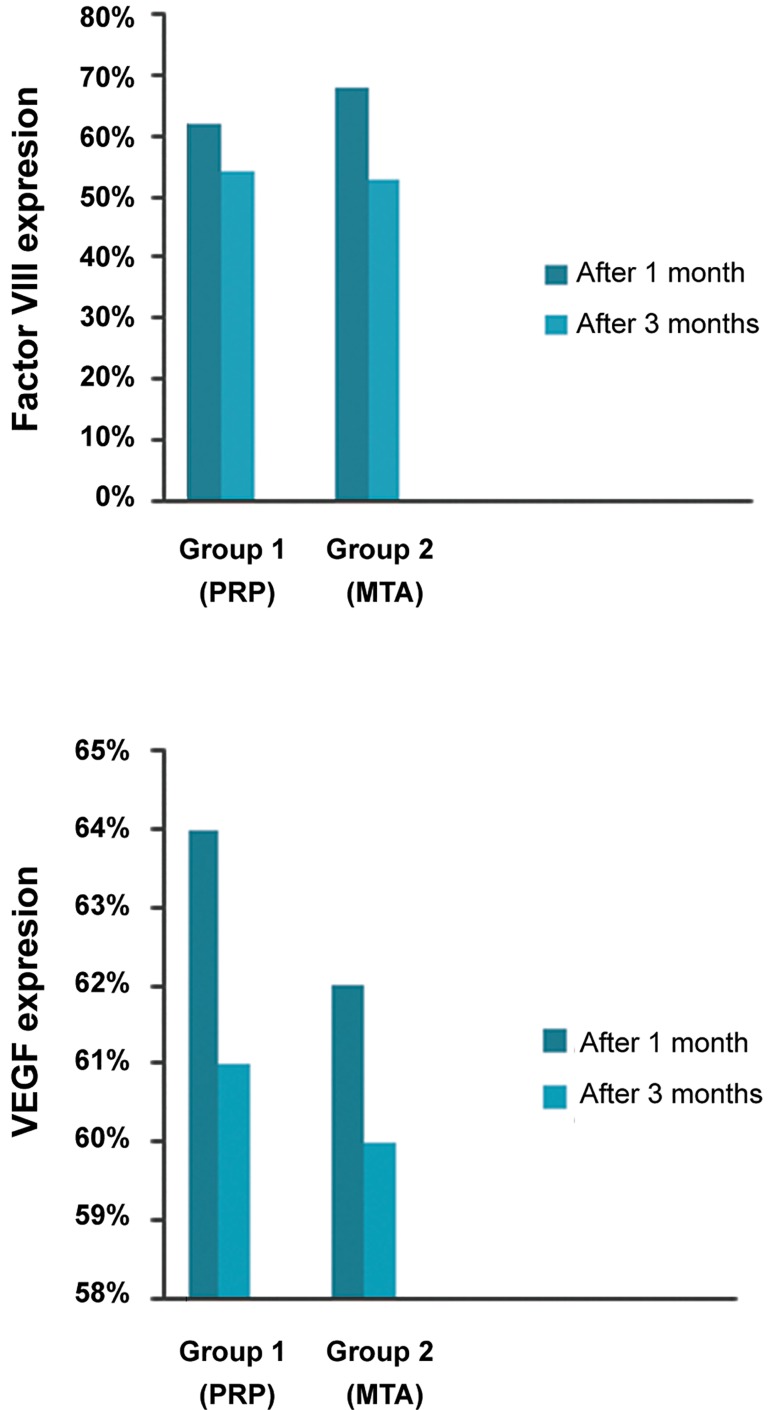
Mean percentage of factor VIII and vascular endothelial growth factor (VEGF) expressions (mean ± SD) in the experimental groups at one and three months after treatment.

Expressions of VEGF and factor VIII were positive in stromal cells and in endothelial cells of the blood vessel walls (Fig.3C,D). Figure 4 shows the mean percentage of VEGF and factor VIII expressions in the experimental groups at one and three months postoperative. Both factors showed downregulation three months postoperative. 

VEGF and factor VIII intensity were severe at one month (I=4), but moderate at three months postoperative in most cases from both groups (I=3, Fig.3C, D). 

## Discussion

Recently, revascularization has been introduced as an acceptable treatment option that may replace the traditional protocols in a necrotic immature tooth with incomplete root development (2,5). Since a matrix is necessary to facilitate cell growth, adhesion, migration, and differentiation, it is recommended to induce bleeding in the canal space in order to form a BC as a matrix. Inability to access the BC has been implicated in unsuccessful results of some regenerative cases (7,16). The PRP contains more than 10^6^ platelets per µL which is five times more than the normal platelet count. PRP possesses a higher concentration of GF such as platelet derived growth factor (PDGF), transforming growth factor β1 (TGFβ1), VEGF, and fibroblast growth factor (FGF) (8). In addition, PRP is capable of restricting inflammation by suppressing cytokines (17). PRP, as a biocompatible and bioactive scaffold, can possibly be joined with the BC to improve the regeneration process. There have been reports of successful PRP application in vascularization of necrotic immature teeth in humans (12,18,19). Following the formation of a BC, Jadhov et al. (20) have applied a PRP soaked collagen sponge and reported enhanced clinical and radiographic outcomes in term of regeneration. However, the present study along with the others, did not demonstrate any improvement in the regeneration of the tissue inside the canal after application of PRP (19,21). Our results showed new tissue formation in 42.8% of the BC+PRP+MTA group and in 43.5% of BC+MTA group. Therefore, no significant association existed between the presence of newly formed tissue and the application of either PRP or a BC as a scaffold. This might be due to the fact that platelet degranulation and release of GFs from PRP has been shown to peaks at approximately 7-14 hours, after which the release of GFs dramatically decreased and its activity might be halted as early as 7-10 days (22,23). It was suggested that continuous release of PRP had superior effects. Kurita et al. placed PRP in a degradable hydrogel where the release of GF could be managed in a controlled way. Therefore, increased revascularization was accomplished (24). 

Thus, it seems that as long as the ideal concentration of platelet has not been achieved, and the standard method of PRP preparation has not been introduced properly, it will be impossible to expect promising results (25). A recent study has been conducted on application of injectable scaffolds combined with alginate microspheresas the carrier of GFs whit the intent to gradually release morphogen signals (26). 

In the present study, although the percentage of the granulation tissue and histologic outcome was similar to other animal studies (27,29), however others attained better outcomes (13,21). In the present study, the pulp tissues with odontoblast cells and dentin were not regenerated in the root canal. This might indicate the destruction of Hertwig’s epithelial root sheet and apical papilla stem cells (30). An animal study showed that, even after 90 days, the vital apical papilla was observed in approximately 67.0% of infected necrotic pulp (31). Thus, the reason for the above mentioned destruction might be the cytotoxicity of the substances used to sterilize the canal system. The concentration of triple antibiotic paste when prepared as thick paste might be detrimental to the survival of apical papilla stem cells (32,33). A high concentration of NaOCl as the root canal irrigation was reported to be harmful for apical papilla stem cells (34). However, induction hemorrhage and GFs activation enabled mesenchymal stem cells to enter the root canal at a rate hundreds of times great than normally found in the systemic blood flow, with subsequent regeneration of new vital tissue. Such stem cells were more likely enter the canal through the periodontal ligament and bone marrow (35). 

In order to have successful regenerative therapy, it is essential to have rapid revascularization of tissue along with the presence of stem cells. Blood vessels provide highly-metabolic regenerating cells with increased demand for nutrients and oxygen. The vessels are also equipped with endothelial cells that have capability to secrete inflammatory mediators that direct immune cells to the site. This is an immunologic reaction required to remove bacterial debris in the necrotic canals. The vessels are in charge of supplying calcium and phosphate ions to mineralize the dentin (26). An investigation on revascularization of the replanted and auto transplanted immature teeth in dogs has shown ingrowth of new vessels during the first few days and angiogenesis in the entire pulp after one month (36). The VEGF is an important regulator of physiologic and pathologic angiogenesis. This regulator be expressed by undifferentiated pulp and endothelial cells in healthy pulp tissues and induce an angiogenic response by enhancement in MVD (37).Following the formation of granulation tissue, VEGF increases from the first day of an inflammatory tissue process, then gradually decreases (38). It has been suggested that dentin matrix also contains VEGF and it may be released from dentin wall of the canal by the demineralization effect of EDTA or medications such as tetracycline (37). 

Reportedly, MTA will increase the secretion of VEGF if it directly contacts the pulp cells and promotes the growth of stem cells via induction signaling molecules (39,40). In the present study, we have shown that both VEGF and factor VIII expressions in perivascular endothelial and stromal cells were strongly positive in granulation tissue in the first month. However, these factors downregulated during the third month following treatment. Therefore, VEGF and factor VIII have played a critical role in initiation of revascularization of the premature necrotic teeth. Expression of these factors by stromal cells is an indication of the acquisition of the capacity for these cells to secrete VEGF and factor VIII as well as the capacity for stem cells to differentiate into endothelial cells which form vascular plexus, possibly through angiogenesis. Thus, most likely the blood supply of granulation tissue inside the canal is not limited to budding of preexisting vessels in periapical tissue (41). The mechanism of revascularization is of great importance to root canals that have a closed anatomy in which the blood vesseles are merely allowed to enter the canal through the apical foramen, particularly in aged teeth where the apical foramen becomes narrower. 

## Conclusion

This study showed that PRP therapy did not influence the results of revascularization treatment. However, further studies would be needed to introduce a standard protocol for PRP preparation with appropriate properties to be used for revascularization treatment. Furthermore, IHC results showed that VEGF and factor VIII played a pivotal role in the formation of new vessels inside the root canals of revascularized immature teeth. The highlighted role of VEGF in revascularization, the ability of stem cells to express VEGF, and obtaining of endothelial cells characteristics *in vivo* may result in further advancement for the design of scaffolds that contain microspheres with capability to carry signaling molecules that encourage rapid revascularization. 
